# Positive health programme for British South Asian women with postnatal depression: a multiperspective qualitative study

**DOI:** 10.1136/bmjopen-2024-096828

**Published:** 2025-12-07

**Authors:** Jahanara Miah, Penny E Bee, Farah Lunat, Rebecca McPhillips, Anna Kathryn Taylor, Saadia Aseem, Deepali Sharma, Nusrat Husain, Carolyn Chew-Graham

**Affiliations:** 1 School of Biology, Medicine and Health, University of Manchester, Manchester, UK; 2 School of Nursing, Midwifery and Social work, University of Manchester, Manchester, UK; 3 Research and Development, Lancashire and South Cumbria NHS Foundation Trust, Preston, UK; 4 Social Care and Society, The University of Manchester, Manchester, UK; 5 Leeds Institute of Health Sciences, University of Leeds, Leeds, UK; 6 Division of Psychology and Mental Health, The University of Manchester, Manchester, UK; 7 The University of Manchester, Manchester, UK; 8 Mental Health Services Oldham, Pennine Care NHS Foundation Trust, Oldham, UK; 9 School of Biology, Medicine and Health, The University of Manchester, Manchester, UK; 10 University of Keele, Keele, UK

**Keywords:** mental health, maternal medicine, community child health, psychosocial Intervention, anxiety disorders, randomized controlled trial

## Abstract

**Abstract:**

**Objectives:**

To explore the views and perspectives of British South Asian (BSA) women and Positive Health Programme (PHP) facilitators on the usefulness and experiences of the PHP intervention for managing postnatal depression (PND) in primary care settings.

**Design:**

Qualitative study with semi-structured interviews to explore perceptions of acceptability and implementation. A patient and public involvement group provided their insights and feedback on study topic guides, analysis and outcomes.

**Setting and participants:**

We sampled trial participants from the PHP intervention database to ensure variation in geographic setting, age, socioeconomic status and ethnicity. PHP facilitators involved in the trial were also invited to participate in an interview.

Interviews with study participants were conducted at participants’ homes, and community centres, or via phone. Interviews with PHP facilitators were conducted via phone or online. Interview recordings were transcribed verbatim and analysed using thematic analysis and subsequently the Theoretical Framework of Acceptability (TFA) was applied. Recruitment took place between February 2017 and March 2020.

**Results:**

Thirty interviews were conducted—19 trial participants and 11 PHP facilitators. The PHP intervention was viewed positively, with appreciation of its therapeutic content and components such as childcare and refreshments that facilitated engagement. Participants reported improved confidence and well-being and supported their needs. Participants understood the intervention’s purpose. Both intervention participants and facilitators noted strengthened self-efficacy.

Some participants experienced difficulties balancing childcare and attendance, implying a need for logistical assistance. Stigma about mental health in the BSA community was viewed as persistent, recommending future programmes efforts on strategies to reduce stigma and develop supportive environment.

**Conclusion:**

This study demonstrates the possibility of PHP intervention being integrated into routine care by providing culturally tailored support for BSA women with PND, primarily through family engagement and facilitator support. Future research on scalability, alongside community engagement efforts, will strengthen its acceptability and broader applicability.

**Trial registration number:**

ISRCTN10697380.

**STRENGTHS AND LIMITATIONS OF THIS STUDY**

- We ensured an active partnership with a patient and public involvement group, which was involved in the study’s design and the discussion of its findings.
- The inclusion of two key stakeholder groups—women of South Asian origin participating in Positive Health Programme (PHP) and PHP facilitators—strengthened the scope of viewpoints captured.
- Applying the Theoretical Framework of Acceptability to the analysis ensured a systematic investigation into acceptability and contributed to a common gap in perinatal mental health research.
- A limitation of this study is that the participants were likely more open to intervention due to being sampled within a trial context.
- This study’s findings may not reflect the experiences of women less likely to seek or engage in such interventions.

## Introduction

 Up to one in five women develop a mental health problem during pregnancy or in the first year after the birth of their baby,^1^ which negatively impacts individual women, infants, families and society.^2^ Ethnicity and culture are considered to have an impact on how and when women seek help for mental health problems during the perinatal period, and many British South Asian (BSA) women are reluctant to approach services for support as they feel the services available are not sensitive to their beliefs.^3^,^5^ Some ethnic minority groups in the UK have a higher burden of mental health disorders, including anxiety and depression, as compared with the majority White population.^6^ There is also a lesser likelihood of these disorders being detected or treated, including during the perinatal period for women from ethnic minority backgrounds.^7^

Women of South Asian origin are at higher risk of developing postnatal depression (PND). A secondary analysis of birth cohort data and linked routine care data in the UK^3^ found an estimated prevalence of between 9.5% and 14% for common mental disorders in British women prebirth. It was found that compared with White British and White Irish populations, the prevalence of PND was significantly higher among South Asian women. A recent report on maternal mental health and well-being during the COVID-19 pandemic, published by the Centre for Mental Health,^8^ addressed the disparity in maternal mental health outcomes caused by the crisis and outlined how the impact has been unequal. The report indicates differences in the perceived adequacy of information among ethnic minority groups during the perinatal period. A lesser number identifying as Asian/British Asian or Black/Black British highlighted that they had less access to the information they needed during pregnancy and postpartum compared with their White counterparts. A significant variation was observed in the proportion of respondents who reported feeling ‘a lot’ more anxious during this time. It was highlighted that 42% of White respondents, 46% of Black/Black British respondents and 50% of Asian/Asian British respondents reported heightened anxiety. Parents aged 25 years or younger (54%) and also those with household incomes below £16 000 (55%) shared similar experiences reporting heightened vulnerability to anxiety. These findings highlight the need for targeted informational and emotional support to address the unique needs of diverse and potentially underserved populations during the perinatal period.

This qualitative study was nested within the ROSHNI-2 study.^9^ This was a multicentre cluster randomised controlled trial (RCT) that evaluated the clinical and cost-effectiveness of the Positive Health Programme (PHP) intervention for PND in British mothers of South Asian origin when compared with treatment as usual. The culturally adapted PHP intervention is based on the principles of cognitive behavioural therapy. Details of culturally adapting the intervention have been described elsewhere.^9 10^ The PHP RCT findings^10 11^ showed a statistically significant reduction in depression severity (Patient Health Questionnaire-9) at the 4-month follow-up for the PHP intervention group. However, this difference was not observed at the 12-month follow-up. The qualitative study, undertaken following the 4-month primary outcome data collection point, aimed to explore the views and perspectives of BSA women and PHP facilitators on the usefulness, experiences and acceptability of the PHP intervention for managing women with PND in primary care settings.

## Methods

### Design

In this qualitative study, we used semi-structured interviews to gather insights from two groups of participants.^11^ The first group consists of BSA women from the intervention arm of the ROSHNI-2 trial (registered with ClinicalTrials.gov under registration number ISRCTN10697380),^11^ including withdrawn participants, and the second group includes PHP facilitators to gather their views on the PHP intervention. Semi-structured interviews explored participant perspectives on the following:

- The acceptability and usefulness of the group intervention from the perspective of BSA women.
- The perspectives of PHP group facilitators about training and delivery of the intervention.

Using the Theoretical Framework of Acceptability (TFA),^12^ which includes dimensions affective attitude, burden, perceived effectiveness, ethicality, intervention coherence, opportunity costs and self-efficacy, the analysis focused on understanding the acceptability of the PHP intervention.

### Setting and participants

A total of 56 participants were purposively selected for interviews, including 32 trial participants, 13 participants who withdrew from the PHP trial and 11 PHP facilitators.

Of the 13 PHP trial participants who withdrew, only two agreed to be interviewed and their insights were included in the analysis to understand their perspectives on reasons for withdrawal from the trial and barriers to engagement. The remaining 11 declined or were unavailable and therefore were excluded.

#### Trial participants

During the baseline assessment, trial participants were provided with information about the qualitative component of the trial. We contacted trial participants who consented to be contacted for a research interview.^10^ We purposively sampled trial participants from the PHP intervention trial database to help ensure a diverse representation from geographic location, age, socioeconomic status and ethnicity. We also invited trial participants who had withdrawn from the intervention to explore the reasons behind their discontinuation. These participants were chosen for their relevance to the research objectives.

Researchers contacted trial participants by telephone or letter to invite them to an interview based on their stated preferences. Those interested were given an information pack, including a participant information sheet (PIS). They were contacted again after approximately 1 week to confirm their willingness to participate.

#### PHP facilitators

PHP facilitators were purposefully selected to ensure a diverse representation across the geographic study sites. To this end, PHP facilitators in the study sites were approached via phone or email to invite them to an interview. Interested individuals received an email with a PIS describing the qualitative study.

Since participants met the inclusion criteria^10 11^ and provided their consent, they were invited to join a semi-structured interview. Trial participants and PHP facilitators were informed that their involvement in the semi-structured interviews was voluntary and that they could withdraw at any time before the data were fully anonymised without any negative consequences. Recruitment continued until the data collected sufficiently addressed the study’s objectives and no new insights emerged.

### Data collection

The recruitment period for this study lasted from February 2017 to March 2020. During the internal pilot phase,^13^ separate topic guides were developed for trial participants (see online supplemental material 1) and PHP facilitators (see online supplemental material 2) and further refined to inform the interview process for the main trial interviews.

#### Trial participants

One-to-one semi-structured interviews were conducted face-to-face (by SA, JB, ZA, AKT) at either the participant’s home, neutral settings such as children’s centres or community centres or via telephone, as per participant preference. A semi-structured topic guide (online supplemental material 1) was used to steer the interview discussion. The topic guides included questions and probes on the context of intervention delivery, study processes and participants’ perspectives on the intervention’s content. With participants’ consent, all interviews were digitally recorded using an encrypted audio-recorder. Interviews with participants ranged from 30 to 60 minutes. For trial participants who did not speak English, interviews were conducted in their preferred language, either with the assistance of an interpreter or a bilingual interviewer. Trial participants were offered a £20 shopping voucher after completing the interview to compensate for their time.

For trial participants who did not speak English, interviews were conducted in their preferred language, either with the assistance of an interpreter or a bilingual interviewer. Interviews conducted in English were transcribed verbatim. For interviews which were conducted in other languages including Urdu or Punjabi, the bilingual research team first translated the recordings into English before transcription. Priority was given to conceptual rather than literal translation and careful attention was made to particularly cultural idioms. Translations were again cross-checked by our bilingual team members with the audio recordings to ensure the meaning remains grounded. Although we did not use formal back-translation, in case of any uncertainties, we discussed within the research team to ascertain the most accurate representation of participants’ intended meaning. Participants did not receive their transcripts for review.

#### PHP facilitators

Interviews with PHP facilitators were conducted (by SA, JB, ZA, AKT) both over the phone and face-to-face in workplace settings, each lasting between 30 and 60 min. The semi-structured topic guide (online supplemental material 2) used for the interviews explored participants’ understanding of PND, their experiences with training and delivering the sessions, their views on the effectiveness of the intervention for BSA women and suggestions for improving engagement and delivery of the intervention. Demographic information was gathered at the end of each interview, and participants were not provided with their transcripts for review.

### Data analysis

An initial thematic analysis was conducted by SA, JB, ZA and RMcP, followed by the use of TFA.^12^ Thematic analysis^14^ was carried out to explore and familiarise with the data by two qualitative researchers (RMcP, DS) who read the transcripts to identify key themes relevant to the study’s aims. Two researchers (RMcP, ZA) independently read transcripts line by line and applied inductive codes to capture key concepts. New codes were developed iteratively; overlapping codes were merged, and distinctions were clarified. The coding framework was discussed in regular meetings with the wider analysis team (SA, JB, ZA, RMcP, DS), allowing comparison of interpretations and resolution of discrepancies. Codes were grouped into categories, developed into themes and then these themes were subsequently mapped onto the domains of the Theoretical Framework of Acceptability (TFA). Recruitment and analysis continued until no substantially new codes or insights were emerging across interviews, which we considered as thematic saturation.

Subsequently, the TFA, which provides a clear formulation and definition of clear constructs related to the acceptability of interventions (table 1), was used to capture responses regarding intervention acceptability from both recipients and those involved in delivering interventions. Using a theoretically drawn framework purposely developed for evaluating the acceptability of healthcare interventions was considered suitable for this study, alongside thematic analysis, to strengthen a multidimensional evaluation of the intervention’s acceptability.

**Table 1:** Theoretical Framework of Acceptability^12^: definition and component of acceptability

Component	Definition
Affective attitude	How an individual feels about the intervention
Burden	The perceived amount of effort that is required to participate in the intervention
Ethicality	The extent to which the intervention has good fit with the individual’s value system
Intervention coherence	The extent to which the participant understands the intervention and how it works
Opportunity costs	The extent to which benefits, profits or values must be given up to engage in the intervention
Perceived effectiveness	The extent to which the intervention is perceived as likely to achieve its purpose
Self-efficacy	The participant’s confidence that they can perform the behaviour(s) required to participate in the intervention

Adapted from Sekhon *et al*.^12^

For the trial participants and PHP facilitators, themes were deductively charted (by SA, JB, ZA, RMcP, DS) into the seven domains of the TFA, across its seven domains to assess the retrospective acceptability of the healthcare intervention from the perspective of those involved. To ensure reliability, all interview transcripts were initially coded in NVivo (QSR International, Warrington, UK) by the first qualitative researcher and then re-coded by the second qualitative researcher. Participant experiences were systematically charted to align with the identified themes and subthemes. Ongoing discussion took place between SA, JB, ZA, RMcP and DS, and with the wider team to reach consensus on the themes, and descriptive quotes included to validate the findings.

### Patient and public involvement and engagement

We set up a patient and public involvement (PPI) group to guide the research team in the different stages of the research study. The PPI group comprised BSA women with lived experiences of PND, including their family members. The PPI group was involved in initial discussions to address challenges related to stigma and accessing mental health services for BSA mothers with postnatal depression. The themes identified by PPI members were incorporated into the topic guides for trial participants. We also consulted with the PPI group to develop the plans for engagement with community members, which contributed to a broader work programme through feedback and input provided via social media and community engagement events. The group contributed to the development of the study materials and interview topic guide. Their guidance helped ensure that the language was culturally sensitive and accessible to South Asian women, highlighting how to phrase questions about mental health and family relationships to minimise stigma. These contributions strengthened the cultural relevance and acceptability of the study materials.

## Results

In total, 30 interviews were conducted, comprising 17 trial participants, two withdrawn participants and 11 PHP facilitators. The demographics of trial participants are detailed in table 2, with details of the PHP facilitators provided in table 3.

**Table 2:** Trial participants’ characteristics (including withdrawn participants)

Characteristics	N=19
Age (years)	
18–25	1
26–35	14
36–45	4
Ethnicity	
Bangladeshi	2
Pakistani	11
Indian	5
Sri Lankan	1
Trial region	
North West England	4
East Midlands	5
London	6
Yorkshire	4

**Table 3:** PHP facilitator characteristics

Characteristics	N=11
Ethnicity	
Bangladeshi	3
Pakistani	6
Indian	2
Trial region	
North West England	1
East Midlands	4
London	5
Yorkshire	1
Educational attainment	
Postgraduate	4
Master’s and postgraduate certificate	3
Competence-based qualification	3
Information missing	1

PHP, Positive Health Programme.

The findings are presented according to the seven domains of the TFA and data extracts provided to illustrate the analysis.

### Affective attitude—how an individual feels about taking part in the intervention

The trial participants shared their initial feelings about the study; they often expressed concerns about confidentiality, fear of judgement from their community and family and anxiety about speaking in a group setting:

I thought people would judge me… I [had] never attended group counselling… everyone else will know your problems and I know they are not supposed to share it, but they might share it. (Trial participant MD-003)

However, despite these initial reservations, most trial participants reported positive attitudes towards the intervention. The therapeutic content and non-therapeutic aspects, such as childcare, refreshments and the chance to connect with other women, were highly valued. The intervention was expressed as life-changing, highlighting the benefits of social interaction and learning valuable skills:

It was a treat honestly… good food, childcare, I made friends learnt some very good things which help me in my daily life…. Truly it was time for me, I will always remember it. Everything was really good. Can’t be thankful enough, these sessions have really changed my life. (Trial participant LO-003)

PHP facilitators of South Asian heritage talked about how they were motivated to join the programme due to its focus on supporting BSA women and addressing maternal mental health issues, which they identified as needing more attention. They saw the intervention as an essential tool for raising awareness of PND and combating stigma within the community. PHP facilitators expressed confidence in delivering the programme, noting that the intervention’s design and manual were well-structured and resonated with the participants’ values:

When I applied for the job, it sorts of appealed to me because it was working with the ethnic minority community. I think that’s an area that we do need to sort of tackle and raise awareness of the stigma that is associated with mental health and especially maternal mental health. So that’s sort of like why I wanted to sort of go forward for the role. (PHP facilitator YO-008)

### Ethicality—the extent to which the intervention has a good fit with an individual’s value system

Many of the trial participants discussed the stigma they perceived in the South Asian community with regard to mental illness in general and PND specifically during their interviews. It was explained, “in our culture, talking about mental health is a bad thing [that] brings a bad name…especially you don’t want to be a bad mom” (trial participant YO-003). However, even in this context, PHP was understood as being a good fit with individual value systems; this was because the sessions “kept my confidentiality but also provided me with right support… these sessions benefitted me a lot” (trial participant YO-004). When asked what sort of help they thought they needed, it was highlighted that “definitely counselling, talking therapy because I didn’t really want to go on medication because I was breastfeeding” (trial participant LO-006).

In the Asian community, it’s a stigma definitely, and even people don’t understand mental health, and they don’t look after it as they do about their physical health… yes, I think they should open up and should tell their family to engage and especially husband and wife can both work together and should support each other. (Trial participant YO-003)

Participants spoke about the significance of the sessions being conducted in the languages spoken by the BSA community:

Most sessions were in English, but sometimes, if anybody did not understand, then they would jump on to Urdu. Rather, we switched from one to another, and it was English, Urdu and Punjabi…. It was all. (Trial participant LO-P003)

They could speak Urdu and English, which was convenient to few other mothers, and it was a help. (Trial participant YO-P003)

Trial participants also appreciated the intervention’s confidentiality and the professional support it offered, which they described as being invaluable alongside family support:

These sessions were beneficial for me that they kept my confidentiality but also provided me with right support that I was looking at that time. I had family support, but I am talking about professional support as I also work in health care, and I know the importance of professional support, so these sessions benefited me a lot. (Trial participant YO-004)

### Intervention coherence—the extent to which the participant understands the intervention, and how the intervention works

The trial participants understood the purpose of PHP before and during the intervention. Many viewed PHP as a tool to help them address their mental health difficulties and improve their problem-solving abilities:

I thought then that I am depressed, and it might help me as I was looking for some support to get me out of my poor situation. (Trial participant LO-003)

It was appreciated that the focus of each session was a different topic, such as assertiveness and self-esteem, which the trial participants found personally beneficial:

Each session was about a different topic… so it explored different things, one I think was assertiveness, saying no to certain things I think that helped me a lot… it was working on yourself, and trying to identify the problems you have to build your self-esteem back up, so that helped yeah. I did work on my self-esteem and also started saying ‘No’ when I wanted to. (Trial participant MD-003)

The PHP facilitators, particularly those who shared similar cultural backgrounds and experiences with the participants, emphasised the importance of the PHP intervention for BSA women. It was felt that the unique challenges faced by the BSA women, particularly those who had recently immigrated and were managing family responsibilities with little support, were addressed by the PHP intervention:

The South Asian British women, who are coming, I mean, from the group we had, I realised that, when mums, mostly mums have come from Bangladesh or India or Pakistan. And then they come here; they don’t have family support over here, so they have to do everything on their own, and there’s already too much on their plate that they have to look after the family. They have to do household chores and then the baby. The mother is already so tired, and there is so much psychological adjustment to motherhood that no one, no one else, is able to understand that. (PHP facilitator LO-009)

PHP facilitators expressed satisfaction with the training they received, which improved their understanding of the intervention and boosted their confidence in delivering it.

### Burden—the perceived amount of effort that is required to participate in the intervention

The effort required to participate in the PHP varied among trial participants and facilitators. Trial participants found balancing attendance with childcare responsibilities challenging, which impacted their ability to attend all sessions: “[they] are too early in the morning. Every mum has to get themselves and their child ready in the morning to go there” (trial participant LO-007). A crèche was provided for children as part of the project delivery, and this was important as trial participants explained, “the creche was an excellent facility. If there was no childcare, I would not have been able to attend honestly” (trial participant YO-P003). However, when trial participants had children who were too old to attend the creche, childcare remained an issue:

I have other kids, and I was concerned about them although they provided the taxi and as well as crèche which was really very good. It was just that I was concerned about my kids, so I became less interested, and this made me skip the sessions. (Trial participant YO-001)

PHP facilitators commented on the group delivery approach and familiarity with the participants making the process smoother for them:

It was not that difficult for facilitating because most of the women who were part of the group were, you know, recruited by myself, sometimes by myself and sometimes by others, and we went to zero visits. For zero visits where you go and invite them, and that part made me familiar with these women and obviously made me confident to go in front of them and obviously facilitate the training. (PHP facilitator LO-001)

However, the PHP facilitators spoke about the effort required when trying to ensure that participants attended the groups and catching them up on things they had missed when they did not attend:

Obviously, you have your challenges that not every mum attends that takes a lot of chasing up and encouragement to attend… One thing I have been doing is obviously a mum has not come for a couple of weeks I feel like it’s more her anxiety of coming to the group that making her not come; then we done a home visit again just to catch them upon sessions… that helps, they end up coming following week. (PHP facilitator LO-009)

Facilitators also talked about the effort that goes into safeguarding participants and the burden they felt around this, particularly concerning a few incidences where PHP attendees mentioned suicidal ideation, “it kind of came up through the group… there was a lot of complicated cases… for a low-level intervention, it was quite overwhelming” (PHP facilitator LO-003).

Some facilitators described the burden associated with listening to participants and becoming “overly involved… it [the first session] was quite upsetting… we did have were mums who were offloading…I did recognise that I was a little bit too involved emotionally” (PHP facilitator LO-002). It was also highlighted that listening to participants prompted some PHP facilitators to think about their own experiences and draw on these to help the women attending the session:

When I was hearing all these things in a session…I was just thinking that when I had a baby in this country, I was so alone… I decided, okay, these are the areas where I'm going to tell the moms that this is what you can do because I’ve been there… I was prepared myself that okay, this is what I might say. (PHP facilitator LO-005)

### Opportunity costs—the extent to which benefits, profits or values must be given up engaging in an intervention

Some trial participants still faced challenges that led them to miss sessions, despite the provision of childcare and transportation to and from the venue and refreshments. PHP facilitators suggested that while individual circumstances such as illness could affect attendance, lack of motivation was also something they observed:

And that’s just down to individual circumstances, you know the babies falling ill or the mums are or falling ill, but there were a few mums who didn’t attend all the sessions, you know when you just can’t be bothered. (PHP facilitator NW-002)

Although PHP facilitators reported that they found it relatively easy to engage and retain women during the intervention, some struggled with a perceived lack of support from the participants’ families, particularly from the heads of households or spouses. This was seen as an additional barrier due to the perceived lack of cooperation from family members affecting attendance and full engagement in the programme.

### Perceived effectiveness—the extent to which the intervention is perceived as likely to achieve (or have achieved) its purpose

Most trial participants viewed the PHP intervention as effective in achieving its intended outcomes:

I am feeling so much better… I got a lot of confidence, and I was a bit hesitant to communicate with other people in public, but I don’t hesitate now… I don’t even fight with my husband anymore, only thing to complain about was I wake early and sleep late. (Trial participant LO-005)

Although the PHP intervention was perceived to have had a positive impact, for some participants experiencing low moods, it was still a struggle:

I feel good, but sometimes you just feel like… you don’t have the support there when sometimes you feel low… it’s made me think how to improve myself, help myself so I think that same time it helped me with my depression so it’s made me do more things…it will take some time obviously to deal with everything but it’s getting better. (Trial participant MD-003)

PHP facilitators also recognised the effectiveness of the intervention, particularly noting that the group format helped participants motivate each other, increase physical activity, boost self-esteem and build trusting relationships:

The research study and the results prove from the feedback of women and the way we noticed changes in them when we see them on the screening and during SCID assessment and on celebrations day, session eleven and celebration day we could tell the difference from majority of moms. (PHP facilitator NW-002)

### Self-efficacy—the participant’s confidence that they can perform the behaviour(s) required to participate in the intervention

After they had taken part in the intervention, trial participants talked about the various behaviours they could now perform as a result and the benefits this had for their well-being. An example was “saying no to things which we don’t want to do without feeling guilty” (trial participant MD-001), which participants felt was important to “give ourselves some time’’ (trial participant LO-002).

Trial participants also discussed the changes they had made to how they managed their time as a result of the behaviours they were encouraged to perform at PHP groups, “the thing I have managed to change in my life is time management; I now get up early with my son at 7, I used to, before attending the sessions, wake up late, sleep like 1–2 am and wake up 10–11, this group has taught me time management” (trial participant LO-005). Participants talked about how they have made changes to their daily activities as a result of PHP:

I think getting rid of the negative emotions and feelings and making positive moves has really changed my daily activity. I do get myself busy in house chores, but sometimes I lose interest, and then I take a tea break to chill out for five minutes and then get back to my work. This keeps me persistent and also enjoy the time that is meant for me only. (Trial participant NW-001)

Prioritising their needs was a behaviour trial participants talked about being able to perform, “there was like a [PHP] session on prioritising my needs, so I prioritise my day and think what’s more important, is cleaning more important or going out more important” (trial participant MD-003), “it was about looking after yourself and taking time out for yourself to keep healthy… So for me, it was… taking a shower and having a cup of tea while my mother looked after my girls, that was enough relaxation for me honestly” (trial participant MD-002).

Many participants talked about applying the ABC (antecedent, behaviour and consequence) model and how this meant “turning all the negative things into positive” (trial participant LO-003). Many described how “the ABC model, in particular, helped me deal with things practically… I do think negatively sometimes but can get rid of them easily now” (trial participant NW-002).

PHP facilitators also highlighted the importance of the training they received, which equipped them to handle the sessions with confidence. Although some PHP facilitators experienced initial apprehension, they described how their confidence grew as they took on more responsibility:

At the beginning I was a bit apprehensive like to run a group because I hadn’t really done that before so I was wondering like would I be able to do it. Like we had the manual as well so we just worked through that and most of the time I was co-facilitator but there were parts when the lead facilitator was off sick and stuff, so I had to do the group on my own. So, I think that really developed my confidence, and I was able to run the programme. (PHP facilitator NW-001)

## Discussion

The TFA shows key insights into the implementation of the PHP intervention and the impact on BSA women with PND. The most supported domains in the PHP intervention were affective attitude, perceived effectiveness, intervention coherence and self-efficacy. Trial participants valued both therapeutic content and non-therapeutic aspects like childcare and social interaction, which increased positive engagement. The success of the PHP intervention also reflects findings from existing research on group-based therapeutic interventions that provide a supportive environment that helps participants practise new skills and improve their self-efficacy.^15 16^ This suggests the PHP intervention could be expanded to other communities facing similar barriers, such as those that have recently immigrated or are part of low-income groups.

The PHP intervention’s culturally sensitive design aligns with the 2014 NICE guideline on antenatal and postnatal mental health and the NHS Long Term Plan,^17^ which emphasise the importance of providing culturally relevant information about perinatal mental health issues, taking into account the fear of stigma and the need for inclusive mental health services for underserved and ethnic minorities. With South Asian communities often facing barriers to mental healthcare due to cultural stigma, interventions like PHP are timely and relevant in increasing engagement and adherence among ethnic minority groups. Also, the implementation of the PHP and participants’ experiences of taking part in the PHP trial highlighted the influence and power dynamics at play within South Asian family structures. Some participants noted the resistance from their husbands or other family members resisting their participation in the programme or limiting their ability to engage fully and how they engaged with the trial. These complex decision-making processes unfold beyond the individual and are shaped by broader sociocultural factors, including cultural norms, gender roles and familial authority. Within families, men or elders often hold more power than women or younger members.^18 19^ There were also concerns raised about confidentiality and stigma in the study, similar to findings from other research on mental health stigma within ethnic minority communities.^20 21^ The provision of culturally tailored interventions helps to address mental health challenges and also aims to reduce stigma. Similar studies using culturally adapted interventions for ethnic minorities have proven effective in both reducing mental health stigma and improving engagement in therapeutic settings.^19 22 23^

Future iterations of the PHP intervention could improve by combining additional parts mainly focused on long-term stigma reduction strategies. Bilateral learning from low- and middle-income countries (LMICs) could be explored to offer valuable insights for reducing mental health stigma in the UK. In many LMICs, innovative, community-based approaches have been developed, such as leveraging local cultural practice and grassroots mental health education to address mental health challenges despite limited resources.^24^ Other studies^21 22 25 26^ in the UK have adapted models such as community engagement, peer support networks^27^ and culturally sensitive interventions, particularly in marginalised or underserved communities where stigma around mental health is still prevalent.

The improvements in self-efficacy reported by participants—particularly in areas like assertiveness and time management—are consistent with Bandura’s theory of self-efficacy,^28^ which emphasises the role of mastery experiences in building confidence. However, there were challenges highlighted in burden and opportunity costs. Difficulties due to childcare and logistical issues, despite the provision of support, were highlighted, as well as barriers due to illness or lack of family cooperation affecting attendance. Notably, in the literature, these issues are also widely documented in community-based interventions,^29^ where external pressures often reduce engagement in interventions. Future development of the PHP could explore more flexible models, including digital or similar to the remote delivery of the PHP intervention delivered during the pandemic.^11 13^ Other studies which have shown promise in maintaining engagement have used digital delivery,^1330^,^33^ particularly for individuals balancing family and health commitments.

In the study, one facilitator reported experiencing emotional stress while delivering the PHP and the emotional stress experienced from supporting or hearing about other people’s traumatic experiences. Although this was not a dominant theme in the findings, it highlights the importance of emotional stress in managing complex cases or safeguarding concerns experienced by PHP facilitators should not be overlooked. Research on burnout and secondary trauma among facilitators working with vulnerable populations^34 35^ highlights the need for ongoing support, staff training, supervision and self-care strategies delivering such interventions.

In addition to PHP facilitators feeling empowered by the comprehensive training they received, which enabled them to deliver the intervention confidently,^36^ sharing similar cultural and linguistic backgrounds with the participants was perceived as an important factor in the intervention’s success. Existing literature has highlighted how peer-led and culturally informed interventions, particularly in mental health settings and culturally competent facilitators, can better build trust and rapport, which is essential for successful interventions in diverse communities.^37 38^

This study makes an important contribution to the literature on culturally adapted CBT interventions for perinatal populations. While previous programmes such as MOMCare^39^ have demonstrated the value of tailoring CBT to address cultural and contextual needs, PHP differs in several key respects. The PHP intervention was adapted in collaboration with South Asian women and facilitators to take into account the cultural and linguistic resonance that extends beyond surface-level adaptations. Second, PHP was delivered in a group format, which fostered peer support and reflected collectivist values common in South Asian family systems.

This qualitative study provides insights into how cultural norms, family dynamics and social expectations shape engagement with perinatal mental healthcare. By capturing the perspectives of both PHP trial participants and PHP facilitators, we are able to understand the knowledge base on group CBT among ethnic minority perinatal populations and highlight the importance of community-driven adaptation.

### Strengths and limitations

A strength of this paper is the team’s approach to gathering perspectives from both the trial participants and PHP facilitators to understand their insight and experience of the PHP intervention. Also, using the TFA of PHP intervention supported the study’s methodological strength to guide the analysis.

A limitation of this paper is that the participants were recruited from within a trial setting for the study. It is plausible that the BSA women included in this study were already more open to the PHP intervention and engaged with the study. The findings may not necessarily reflect the experiences or perspectives of BSA women who are more opposed to or less likely to seek intervention or are less inclined to participate in research or seek help. An important limitation to note is that of the 13 PHP trial participants who withdrew from the trial and were invited to take part in the interviews, only two agreed and 11 declined or were unavailable to take part in the interview; therefore, their experiences are not reflected in the findings. This has implications for potentially biased results that favour those who have had positive experiences.

Although the predominant themes in the findings were positive, it is worth noting that this could be influenced by factors such as the gratitude bias, where participants may have wanted to appear thankful or exhibited social desirability, providing answers they felt were expected or acceptable, particularly in this study, where the participants shared similar cultural and linguistic backgrounds with the interviewers. The study’s focus on a specific cultural group—BSA women—means the findings may not be directly transferable to other cultural groups or settings.

Although the targeted sampling approach effectively engaged PHP facilitators who were experts in delivering the intervention, it may have introduced a selection bias. The facilitators involved in the study were likely the most engaged and experienced, which could mean that the findings reflect a more positive view of the intervention than might be the case with a broader or more varied sample of facilitators. The PHP facilitators’ experiences captured in this study in implementing the PHP intervention may partially represent the challenges and barriers but not demonstrate the experiences that could arise in different contexts or with less experienced facilitators. Another point to note is that some PHP facilitators were known to the researchers through their professional interactions in the study. The familiarity with the PHP facilitators may have introduced biases in the responses and the data collection process and may have impacted the authenticity of the feedback provided.

This qualitative study captures the short-term, self-reported experiences of the participants, which provide valuable insights into their immediate perceptions of PHP intervention. Despite these insights, we cannot be sure that the reported benefits were sustained over time or translated into meaningful long-term changes in behaviour, well-being or family dynamics, without long-term follow-up. To assess the durability and broader impact of the PHP, future research incorporating longitudinal designs and objective outcome measures would address this understanding.

### Reflexivity and researcher positionality

The research team for this study involved mainly female researchers of South Asian backgrounds and qualitative research, some of whom shared cultural and linguistic backgrounds with participants they interviewed. It is possible that this shared identity supported rapport-building and facilitated open discussion, particularly on sensitive topics such as perinatal mental health. Counteracting this, it may also have influenced how participants chose to present their experiences and presented an element of bias by providing culturally acceptable narratives of PHP.

As mentioned in the earlier text, a small number of PHP facilitators were known to members of the research team prior to the study. To manage this, we explicitly acknowledged professional boundaries at the outset of the interviews and maintained a neutral, non-judgmental stance during data collection. The team acknowledged that these prior relationships might have influenced responses from PHP facilitator participants, which could have encouraged openness due to trust; however, this could have also introduced the possibility of social desirability bias. These influences were discussed during the analysis to ensure transparency and reflexivity in interpretation.

## Conclusion

For future development of the PHP intervention for BSA women with PND, building on particular logistical support, extending family engagement efforts and addressing the emotional strain on facilitators could improve the intervention’s acceptability and effectiveness. Also, alongside the PHP intervention, there is a need for active community engagement efforts to reduce stigma around mental health within the BSA community, which remains significant to promoting a supportive environment.

The constructive reception of the PHP intervention highlights the possibility of such models being incorporated into routine care. The literature is increasingly recognising that community-based and culturally tailored interventions should be integrated into standard mental healthcare to better serve diverse populations.^19 40 41^ Future studies could examine the scalability of the PHP intervention and its adaptability to other ethnic minority groups and foster targeted interventions that address cultural and systemic barriers to care.

## Supplementary material

10.1136/bmjopen-2024-096828 online supplemental file 1

10.1136/bmjopen-2024-096828 online supplemental file 2

## Data Availability

Data are available on reasonable request.
