# Supplementary material for: Positive health programme for British South Asian women with postnatal depression: a multiperspective qualitative study
Source: BMJ Open. 2025 Dec 7;15(12):e096828. doi: 10.1136/bmjopen-2024-096828 (PMC12684183; doi:10.1136/bmjopen-2024-096828)
Supplement: online supplemental file 1 [file bmjopen-15-12-s001.docx]

**Topic Guide - Trial participants - supplementary material 1**

**1) Opening**

Explain the study and the aims of the research

Explain the purpose of the interview

Clarify confidentiality and check consent

Invite and answer any questions

1. Can you tell me how you were invited to participate in the ROSHNI2 study? (explore whether letter received, suggestion from GP/HV)
   1. Did you think this study was for you? Why/why not?
   2. What information did the researcher give you?
   3. What would you think if you were not allocated to the PHP group?
   4. Will this in any way make you feel that you are not worthy……?
   5. What did other people think when you were invited to participate in this study? (explore husband, family, other)
   6. Was it your own decision to take part in the study? If yes/No why?
2. Please can you tell me about yourself and your family>
3. Can you describe you problems……
4. What do you understand by ‘depression’?
5. Did you think you might have low mood or ‘depression’?

b. Had you discussed your problems with anyone (explore family, husband, GP, HV, other)

- 1. Did other people think you might be depressed?
  2. What sort of help did you think you needed?
  3. What sort of help did other people like your husband/mother in law think you needed?
  4. What is generally the perception of South Asian community about depression?
  5. Do you think there is bad name/stigma attached to mental health?
  6. If yes how can we bring more awareness about mental health in the South Asian community?

1. After seeing the researcher, why did you agree to join the study?
   1. Did you talk to anyone about your decision to participate in the study?
   2. What did other people think about your taking part in the study?
   3. Was there anything in particular that persuaded you to take part?
   4. What were your expectations?
2. How did you find the group?
   1. What was helpful? Why?
   2. What solutions did you take from the group sessions?
   3. Which were the best parts of the group sessions?
   4. Was there anything that was not very helpful?
   5. How did you relate yourself to the group leader?
   6. How did you find being in a group with other women?
   7. How many sessions did you go to?
      1. If you didn’t go to all 12 sessions – why?
3. How do you feel now?
   1. How did the group help?
   2. What skills do you think that you took from the sessions?

do you feel able to manage your mood and feelings now?

- 1. What impact is that having on your daily life/
  2. What really did you learn from PHP sessions?
  3. In what way has it really helped you in coping with your depression?
  4. Do you see any of the other women now?
  5. Is there anything else that could have been done?
  6. Would you recommend the group to anyone else?
  7. What sort of people would most enjoy attend the group?
  8. What sort of people would not enjoy attending the group?
