# Supplementary material for: Positive health programme for British South Asian women with postnatal depression: a multiperspective qualitative study
Source: BMJ Open. 2025 Dec 7;15(12):e096828. doi: 10.1136/bmjopen-2024-096828 (PMC12684183; doi:10.1136/bmjopen-2024-096828)
Supplement: online supplemental file 2 [file bmjopen-15-12-s002.docx]

**Topic Guide - PHP facilitators - supplementary material 2**

***1) Opening***

*Explain the study and the aims of the research*

*Explain the purpose of the interview*

*Clarify confidentiality and check consent*

*Invite and answer any questions*

1. Can you tell me why you participated in this study?
2. Can you tell me what you understand by the term ‘post-natal depression’?
   1. How can PND affect women of SA origin?
   2. Are there any barriers to SA women seeking help for PND? What are they?
   3. How can these best be overcome?
   4. Are there any enablers to SA women seeking help?
3. What do you feel about the group sessions in ROSHNI2 – how helpful is this intervention for SA women with PND?

a. Why?

b. What are the most valuable components?

C. can you comment on the content of each session;

**Positive Health Program (12 sessions)**

**Session 1: Introduction to the Positive Health Programme**

**Session 2- Pressures and expectations of being a woman**

**Session 3 – Understanding and managing self-esteem (Part 1)**

**Session 4 – Understanding and managing self-esteem (Part 2)**

**Session 5: Assertiveness and confidence building**

**Session 6: “Keeping up with the Joneses”**

**Session 7 – Religion & Spirituality**

**Session 8: Breaking Social Isolation & Building Social Networks**

**Session 9 – Exercise, Looking Good and Building Motivation**

**Session 10 – Relaxation: “Taking time out”**

**Session 11 – Discussion (Participant choice)**

**Session 12 – Award Ceremony and Party**

1. How did you find the training?
   1. What were the best parts?
   2. What could have been done differently?
   3. What skills did you develop through the training?
   4. How well do you remember the training now?
2. How did you find delivering the sessions?
   1. What went well? Can you tell me why?
   2. What went less well? Can you tell me why?
3. What has it been like working on a trial? (*Explore experiences of delivering sessions to a protocol). Do you think it would be different delivering the intervention in practice? How/why?*
4. Can you talk through your experiences of supervision? (*Explore what sort of things they discussed). What was helpful? What was less helpful? Do you think the supervision is useful? What could be done to help people get more from supervision?*
5. What would help people like you deliver the intervention?
6. What would help women attend and engage with the intervention?
