# Supplementary material for: Positive health programme for British South Asian women with postnatal depression: a multiperspective qualitative study
Source: BMJ Open. 2025 Dec 7;15(12):e096828. doi: 10.1136/bmjopen-2024-096828 (PMC12684183; doi:10.1136/bmjopen-2024-096828)
Supplement: Supplementary file 3 [file bmjopen-2024-096828.reviewer_comments.pdf]

PEER REVIEW HISTORY

BMJ Open publishes all reviews undertaken for accepted manuscripts. Reviewers are asked to complete a checklist review form and are provided with free text boxes to elaborate on their assessment. These free text comments are reproduced below.

ARTICLE DETAILS

Title (Provisional)

Positive Health Programme for postnatal depression women of South Asian Origin: a multi-perspective qualitative study

Authors

Miah, Jahanara; Bee, Penny E; Lunat, Farah; McPhillips, Rebecca; Taylor, Anna Kathryn; Aseem, Saadia; Sharma , Deepali; Husain, Nusrat; Chew-Graham, Carolyn

---

VERSION 1 - REVIEW

**Reviewer**	**1**
**Name**	**Borghei, Narjes Sadat**
**Affiliation**	**Mashad University of Medical Science, Nursing & Midwifery faculty**
**Date**	**04-Jan-2025**
**COI**	**None**

Hi my colleges editor of chief

Thank you for choosing me as a reviewer.

It is a good article. Below I will send my suggestions to improve the quality of the

1-The abstract is very long (393 word) please please make it shorter.

2-Which method did the researcher used for processing data prior to analysis,including transcription, data entry, data management and security, verification of data integrity, data coding?

3- which Techniques did the researcher used for enhance trustworthiness and credibility of data analysis?

4- Please clarify the researcher about Process by which inferences and themes. I think it didn't identified .

thanks

**Reviewer**	**2**
**Name**	**Kossakowska, Karolina**
**Affiliation**	**University of Lodz, Institute of Psychology**
**Date**	**19-Jul-2025**
**COI**	**None**

Dear Authors,

Thank you for the opportunity to review your manuscript. The topic you address is highly relevant and timely, as it concerns culturally sensitive care for British South Asian (BSA) women experiencing postnatal depression (PND), a population often underserved by mainstream mental health services. The inclusion of both participant and facilitator perspectives is a clear strength of the study, and the use of the Theoretical Framework of Acceptability (TFA) provides a potentially useful structure for understanding intervention feasibility and reception.

However, in its current form, the manuscript bears certain features more typical of an internal evaluation report than of a fully developed qualitative research article. Some key methodological details are underreported, the analytic depth could be significantly enhanced, and the findings appear predominantly positive, raising concerns about possible confirmation bias or limited exploration of disconfirming cases. I therefore recommend a major revision.

Below I outline specific areas that require attention:

1. While purposive sampling is noted, the rationale for inclusion and exclusion – particularly for the 13 withdrawn participants (of whom only 2 were ultimately interviewed) – should be clarified (p. 6, lines 154–165 and then p. 10, lines 256-258). Were the views of those two women included in the results?

2. While the manuscript includes an information about the facilitators' cultural and professional backgrounds, it would be important to also reflect on the positionality of the interviewers and their relationships with participants or facilitators. In particular, the manuscript notes that some facilitators were „known to the researchers" (p. 23, lines 607–610), which may have shaped participants' responses or influenced the tone of the interviews. A brief reflexive statement addressing how this familiarity was managed and interpreted would strengthen the transparency of the study.

3. Some interviews were conducted in Urdu or Punjabi and translated into English (p. 8, lines 197–201), but there is no detail on how meaning was preserved, whether back-translation was used, or whether cultural idioms were checked for equivalence.

4. The thematic analysis is presented in broad strokes. The process by which codes were developed, refined, and grouped into themes is not detailed (pp. 9-10, lines 239–242). What was the coding framework? Were codes discussed collaboratively among authors? Was saturation tracked?

5. The use of the TFA framework sometimes appears overly rigid. For example, the quote on p. 12, lines 285–288 („It was a treat honestly...”) is placed under „Affective Attitude" but not examined further. Consider reflecting on the psychological mechanisms implied in such statements.

6. You mention the involvement of a PPI group which is an important asset. However, the manuscript could elaborate on how their contributions shaped the topic guide.

7. The findings are strongly skewed toward positive interpretations. Were any disconfirming experiences or frustrations reported but omitted? This risks introducing confirmation bias. If all participants spoke highly of the programme, the authors should explain this phenomenon (e.g., gratitude bias, social desirability).

8. As you reported, one facilitator acknowledges emotional strain (p. 17, lines 419–423) yet the potential for vicarious trauma or burnout is not explored in the discussion.

9. Power dynamics within South Asian family systems (e.g., mention of resistance from husbands or other family members) could also be analyzed more critically to show how PHP interacts with complex sociocultural factors.

10. The limitations section could be expanded to include:

• Lack of representation from those who disengaged.

• Prior relationships between facilitators and researchers.

• Absence of long-term outcome data (beyond self-reported experiences).

11. The manuscript would benefit from more clearly stating its original contribution. How does PHP differ from other culturally adapted CBT interventions (e.g., MOMCare)? What does this study add to the knowledge base on group CBT among ethnic minority perinatal populations?

12. Consider briefly discussing the scalability of PHP, especially since remote adaptation is mentioned (p. 22, lines 568–575), but not developed.

13. Minor or Technical Issues

• Article title: The current title („Positive Health Programme for postnatal depression women…”) contains a grammatical error – I suggest changing it to: „Positive Health Programme for women with postnatal depression...” or „…for South Asian women with postnatal depression”.

• Page 7, line 202: the authors write „...they were first translated into English and then transcribed verbatim" – it's unclear who did the translations and how their quality was ensured.

• Page 10, lines 239–240: In the sentence „Participant experiences were systematically charted to align…", it would be helpful to clarify what „systematically charted" specifically refers to (e.g., NVivo matrix, manual charting, etc.)

• Page 22, lines 607–610: Already noted earlier – the familiarity between researchers and facilitators should be more fully addressed, as it may have influenced data collection (e.g., expectancy effect, limitations in openness, etc.).

VERSION 1 - AUTHOR RESPONSE

Reviewer: 1

Dr. Narjes Sadat Borghei, Mashad University of Medical Science, Golestan University of Medical Science

Comments to the Author:

It is a good article. Below I will send my suggestions to improve the quality of the

The abstract is very long (393 word) please please make it shorter.	Thank you very much for taking the time to review our work and we appreciate your constructive suggestions to help strengthen and improve the manuscript.<br><br>Word count in the abstract is now reduced to 299 (P.2) and reads as:<br><br>"Abstract<br><br>Objectives: To explore the views and perspectives of British South Asian (BSA) women and Positive Health Programme (PHP) facilitators on the usefulness and experiences of the PHP intervention for managing postnatal depression (PND) in primary care settings.<br><br>Design: Qualitative study with semi-structured interviews to explore perceptions of acceptability and implementation. A Patient and Public Involvement group provided their insights and feedback on study topic guides, analysis, and outcomes.<br><br>Setting and participants: We sampled trial participants from the PHP intervention database to ensure variation in geographic setting, age,

	socioeconomic status, and ethnicity. PHP facilitators involved in the trial also invited to participate in an interview.<br><br>Interviews with study participants were conducted at participants' homes, and community centres, or via phone. Interviews with PHP facilitators were conducted via phone or online. Interview recordings were transcribed verbatim and analysed using thematic analysis and subsequently the Theoretical Framework of Acceptability (TFA) was applied. Recruitment took place between February 2017 and March 2020.<br><br>Results: Thirty interviews were conducted – 19 trial participants and 11 PHP facilitators. The PHP intervention was viewed positively, with appreciation of its therapeutic content and components such as childcare and refreshments facilitated engagement. Participants reported improved confidence and well-being and supported their needs. Participants understood the intervention's purpose. Both intervention participants and facilitators noted strengthened self-efficacy.<br><br>Some participants experienced difficulties balancing childcare and attendance, implying a need for logistical assistance. Stigma about mental health in the BSA community was viewed as persistent, recommending future programs efforts on strategies to reduce stigma and develop supportive environment.<br><br>Conclusion: This study demonstrates the possibility of PHP intervention being integrated into routine care by providing culturally tailored support for BSA women with PND, primarily through family engagement and facilitator support. Future research on scalability, alongside community engagement efforts will strengthen its acceptability and broader applicability.”
Which method did the researcher used for processing data prior to analysis,including transcription, data entry, data	Thank you for raising these points:<br><br>The English interviews were transcribed verbatim. Non-English interviews were conducted in participants’ preferred language. They were

management and security, verification of data integrity, data coding?	translated into English first and then transcribed verbatim. Transcription was done by the research team/interviewers, and not outsourced to a professional service.<br><br>Data entry<br>Transcripts were uploaded and stored in NVivo (QSR International) for qualitative coding and analysis.<br><br>Data management and security<br>Interviews were digitally recorded on audio recorders. Data were stored securely with confidentiality maintained. Identifiable information was anonymised (participants were identified with ID codes) and only the research team had access, with restricted, secure storage.<br><br>Verification of data integrity<br>Non-English interviews were translated into English before transcription. Double-coding was done and transcripts were coded by one qualitative researcher, then re-coded independently by a second researcher. Cross-checking was done via team discussions (SA, JB, ZA, RM, DS) to resolve differences and refine themes. This iterative process ensured accuracy, completeness, and reliability.<br><br>Data coding<br>The approach used for data coding was a combination of inductive thematic analysis (to identify themes emerging from data) and deductive coding using the Theoretical Framework of Acceptability (TFA). NVivo was used to organise and manage codes. Multiple researchers coded transcripts; disagreements were resolved through discussion until consensus was reached. Themes were refined and mapped into the 7 domains of the TFA (e.g., Affective Attitude, Burden, Self-Efficacy).<br><br>We have altered this to now read as (P.8):<br><br>"An initial thematic analysis was conducted by SA, JB, ZA, RM, followed by the use of TFA (12). Thematic analysis (14) was carried out to explore and familiarise with the data by two qualitative researchers (RM, DS) who read the transcripts to

	identify key themes relevant to the study's aims. Two researchers (RM, ZA) independently read transcripts line by line and applied inductive codes to capture key concepts. New codes were developed iteratively; overlapping codes were merged, and distinctions were clarified. The coding framework was discussed in regular meetings with the wider analysis team (SA, JB, ZA, RM, DS), allowing comparison of interpretations and resolution of discrepancies. Codes were grouped into categories, developed into themes and then these themes were subsequently mapped onto the domains of the Theoretical Framework of Acceptability (TFA). Recruitment and analysis continued until no substantially new codes or insights were emerging across interviews, which we considered as thematic saturation.”
which Techniques did the researcher used for enhance trustworthiness and credibility of data analysis?	Thank you for this question:<br><br>We used several strategies to improve the trustworthiness and credibility of the analysis. These included double-coding, ensuring each transcript was coded by one researcher and then independently re-coded by a second researcher. Codes and emerging themes were reviewed and refined through iterative team discussions (SA, JB, ZA, RM, DS) until consensus was reached.<br><br>The approach used for deductive coding was guided by the Theoretical Framework of Acceptability (TFA), for a transparent and theoretical structure for mapping themes. As we used thematic analysis to inductively capture participants’ voices, while deductive coding ensured alignment with established domains of acceptability. NVivo software was used to manage transcripts, codes, and memos, enabling systematic organisation and traceability of analytic decisions. For non-English interviews, translation into English was followed by transcription and then cross-checking within the team to ensure accuracy and preserve meaning.<br><br>We have altered this to now read on (P.8):<br><br>“An initial thematic analysis was conducted by SA, JB, ZA, RM, followed by the use of TFA (12). Thematic

	analysis (14) was carried out to explore and familiarise with the data by two qualitative researchers (RM, DS) who read the transcripts to identify key themes relevant to the study's aims. Two researchers (RM, ZA) independently read transcripts line by line and applied inductive codes to capture key concepts. New codes were developed iteratively; overlapping codes were merged, and distinctions were clarified. The coding framework was discussed in regular meetings with the wider analysis team (SA, JB, ZA, RM, DS), allowing comparison of interpretations and resolution of discrepancies. Codes were grouped into categories, developed into themes and then these themes were subsequently mapped onto the domains of the Theoretical Framework of Acceptability (TFA). Recruitment and analysis continued until no substantially new codes or insights were emerging across interviews, which we considered as thematic saturation."
Please clarify the researcher about Process by which inferences and themes. I think it didn't identified .	Thank you for this comment:<br><br>As mentioned above (P.8), the themes and inferences were identified through an iterative process involving familiarisation with transcripts, inductive and deductive coding in NVivo, and mapping to the Theoretical Framework of Acceptability (TFA). Double-coding and team discussions ensured consistency, with codes refined into broader categories and then developed into themes grounded in participants' accounts.

thanks

Reviewer: 2

Dr. Karolina Kossakowska, University of Lodz

Comments to the Author:

Dear Authors,

Thank you for the opportunity to review your manuscript. The topic you address is highly relevant and timely, as it concerns culturally sensitive care for British South Asian (BSA) women experiencing postnatal depression (PND), a population often underserved by mainstream mental health services. The inclusion of both participant and facilitator perspectives is a clear strength of the study, and the use of the Theoretical Framework of Acceptability (TFA) provides a potentially useful structure for understanding intervention feasibility and reception.

However, in its current form, the manuscript bears certain features more typical of an internal evaluation report than of a fully developed qualitative research article. Some key methodological details are underreported, the analytic depth could be significantly enhanced, and the findings appear predominantly positive, raising concerns about possible confirmation bias or limited exploration of disconfirming cases. I therefore recommend a major revision.

Below I outline specific areas that require attention:

1. While purposive sampling is noted, the rationale for inclusion and exclusion – particularly for the 13 withdrawn participants (of whom only 2 were ultimately interviewed) – should be clarified (p. 6, lines 154–165 and then p. 10, lines 256-258). Were the views of those two women included in the results?	Thank you very much for taking the time to review our work and we appreciate your constructive suggestions to help strengthen and improve the manuscript.<br><br>We have altered this to now read on (P. 5) as:<br><br>"A total of 56 participants were purposively selected for interviews, including 32 trial participants, 13 participants who withdrew from the PHP trial, and 11 PHP facilitators.<br>Of the 13 PHP trial participants who withdrew, only 2 agreed to be interviewed and their insights were included in the analysis to understand their perspectives on reasons for withdrawal from the trial and barriers to engagement. The remaining 11 declined or were unavailable, and therefore were excluded."
2. While the manuscript includes an information about the facilitators' cultural and professional backgrounds, it would be important to also reflect on the positionality of the interviewers and their	Thank you for this comment. Based on your suggestion, we have now included a section on Reflexivity and Researcher Positionality on (P.22), which r**eads as:**

relationships with participants or facilitators. In particular, the manuscript notes that some facilitators were „known to the researchers" (p. 23, lines 607–610), which may have shaped participants' responses or influenced the tone of the interviews. A brief reflexive statement addressing how this familiarity was managed and interpreted would strengthen the transparency of the study.	"The research team for this study involved mainly female researchers of South Asian backgrounds and qualitative research, some of whom shared cultural and linguistic backgrounds with participants they interviewed. It is possible that this shared identity supported rapport-building and facilitated open discussion, particularly on sensitive topics such as perinatal mental health. Counteracting this, it may also have influenced how participants chose to present their experiences, and presented an element of bias by providing culturally acceptable narratives of PHP.<br>As mentioned in the earlier text, a small number of PHP facilitators were known to members of the research team prior to the study. To manage this, we explicitly acknowledged professional boundaries at the outset of the interviews and maintained a neutral, non-judgmental stance during data collection. The team acknowledged that these prior relationships might have influenced responses from PHP facilitator participants, which could have encouraged openness due to trust; however, this could have also introduced the possibility of social desirability bias. These influences were discussed during the analysis to ensure transparency and reflexivity in interpretation."
3. Some interviews were conducted in Urdu or Punjabi and translated into English (p. 8, lines 197–201), but there is no detail on how meaning was preserved, whether back-translation was used, or whether cultural idioms were checked for equivalence.	Thank you for this question:<br><br>Interviews conducted in Urdu or Punjabi were translated into English prior to transcription. To ensure that meaning was preserved interviews were conducted by bilingual researchers fluent in English and the relevant South Asian language. Translations prioritised conceptual and cultural equivalence rather than literal word-for-word translation. Particular attention was given to idiomatic expressions. Where ambiguity arose, bilingual members of the research team (SA, ZA) discussed the phrasing to agree on the most accurate representation of participants' intended meaning.

	While we did not have formal back translation, our bilingual researchers cross-checked transcripts against the audio recordings. This served to safeguard accuracy and minimise risk of distortion.<br><br>We have revised the Methods section to clarify these steps and acknowledge the absence of formal back-translation as a limitation (P.7) this reads now as:<br><br>"For trial participants who did not speak English, interviews were conducted in their preferred language, either with the assistance of an interpreter or a bilingual interviewer. Interviews conducted in English were transcribed verbatim. For interviews which were conducted in other languages including Urdu or Punjabi, the bilingual research team first translated the recordings into English before transcription. Priority was given to conceptual rather than literal translation and careful attention was made to particularly cultural idioms. Translations were again cross-checked by our bilingual team members with the audio recordings to ensure the meaning remains. Although we did not use formal back-translation, in case of any uncertainties, we discussed within the research team to ascertain the most accurate representation of participants' intended meaning. Participants did not receive their transcripts for review."
4. The thematic analysis is presented in broad strokes. The process by which codes were developed, refined, and grouped into themes is not detailed (pp. 9-10, lines 239–242). What was the coding framework? Were codes discussed collaboratively among authors? Was saturation tracked?	Thank you for this question:<br><br>The process followed for thematic analysis and coding process was as below:<br>Initial coding: Two researchers (RM, ZA) independently read transcripts line by line and applied inductive codes to capture salient concepts.<br>Code refinement: New codes were developed iteratively; overlapping codes were merged, and distinctions were clarified.

	Collaborative discussion: The coding framework was discussed in regular meetings with the wider analysis team (SA, JB, ZA, RM, DS), allowing comparison of interpretations and resolution of discrepancies.<br>Theme development: Codes were grouped into categories and then developed into themes. Themes were subsequently mapped onto the domains of the Theoretical Framework of Acceptability (TFA).<br>Saturation: Recruitment and analysis continued until no substantially new codes or insights were emerging across interviews, which we interpreted as thematic saturation.<br><br>We have added these details to the Data Analysis section for clarity (P.8)<br><br>"An initial thematic analysis was conducted by SA, JB, ZA, RM, followed by the use of TFA (12). Thematic analysis (14) was carried out to explore and familiarise with the data by two qualitative researchers (RM, DS) who read the transcripts to identify key themes relevant to the study's aims. Two researchers (RM, ZA) independently read transcripts line by line and applied inductive codes to capture key concepts. New codes were developed iteratively; overlapping codes were merged, and distinctions were clarified. The coding framework was discussed in regular meetings with the wider analysis team (SA, JB, ZA, RM, DS), allowing comparison of interpretations and resolution of discrepancies. Codes were grouped into categories, developed into themes and then these themes were subsequently mapped onto the domains of the Theoretical Framework of Acceptability (TFA). Recruitment and analysis continued until no substantially new codes or insights were emerging across interviews, which we considered as thematic saturation."
5. The use of the TFA framework sometimes appears overly rigid. For example, the quote on p. 12, lines 285–288 („It was a treat honestly...") is placed under	Thank you for this comment:

„Affective Attitude" but not examined further. Consider reflecting on the psychological mechanisms implied in such statements.	We used the TFA framework as a structured approach for understanding intervention acceptability while remaining grounded in participants' responses. In some cases, such as the quote ("It was a treat honestly…"), we acknowledge that the richness of psychological mechanisms (e.g., experiences of selfcare, respite, and validation) could have been more explicitly done. At the same time, we recognise that deeper interpretation carries the risk of diverging from the participant's intended meaning, particularly given the role of translation in some interviews.
6. You mention the involvement of a PPI group which is an important asset. However, the manuscript could elaborate on how their contributions shaped the topic guide.	Thank you for recognising this, we have now included on how the PPI groups contributions shaped the study materials (P.9). Specifically, on the language and cultural relevance of the topic guide, suggesting that we frame sensitive questions around mental health and family relationships in ways that would be more comfortable for South Asian women to discuss.<br><br>This now reads as:<br>"The group contributed to the development of the study materials and interview topic guide. Their guidance helped ensure that the language was culturally sensitive and accessible to South Asian women. Highlighting how to phrase questions about mental health and family relationships to minimize stigma. These contributions strengthened the cultural relevance and acceptability of the study materials."
7. The findings are strongly skewed toward positive interpretations. Were any disconfirming experiences or frustrations reported but omitted? This risks introducing confirmation bias. If all participants spoke highly of the programme, the authors should explain this phenomenon (e.g., gratitude bias, social desirability).	Thank you for this suggestion:<br><br>All participant responses were analysed through thematic analysis, and all feedback was considered in the study; no recurring negative themes emerged. Although the predominant themes were positive, there were a few disconfirming experiences, such as skipping sessions due to participants who had older children, the creche didn't address that need

	and due to lack of family support, also observation from PHP facilitators on the lack of motivation for some trial participants in attending the sessions due to lack of motivation (P.17) were reported. Although the findings reflect genuine participant satisfaction, as you have highlighted, it could also be influenced by factors such as gratitude bias, and we have now included this in our discussion (P.23). This now reads as:<br><br>“Though the predominant themes in the findings were positive, it is worth noting that this could be influenced by factors such as the gratitude bias, where participants may have wanted to appear thankful or exhibited social desirability, providing answers they felt were expected or acceptable, particularly in this study, where the participants shared similar cultural and linguistic backgrounds with the interviewers. The study's focus on a specific cultural group - BSA women - means the findings may not be directly transferable to other cultural groups or settings.”
8. As you reported, one facilitator acknowledges emotional strain (p. 17, lines 419–423) yet the potential for vicarious trauma or burnout is not explored in the discussion.	We have included more on emotional strain in the discussion section, focusing on the potential challenges for facilitators (P.21). This now reads as:<br><br>“In the study one facilitator reported experiencing emotional stress while delivering the PHP and the emotional stress experienced from supporting or hearing about other people’s traumatic experiences. Although this was not a dominant theme in the findings, it highlights the importance of emotional stress in managing complex cases or safeguarding concerns experienced by PHP facilitators should not be overlooked. Research on burnout and secondary trauma among facilitators working with vulnerable populations (34, 35) highlights the need for ongoing support, staff training, supervision, and self-care strategies delivering such interventions”

9. Power dynamics within South Asian family systems (e.g., mention of resistance from husbands or other family members) could also be analyzed more critically to show how PHP interacts with complex sociocultural factors.	Thank you for this suggestion, we have included this point further in the discussion section on exploring how cultural and family power structures influence the program's implementation and participants' experiences, rather than just noting that resistance exists (P.20), this now reads as:<br><br>"Also, the implementation of the PHP and participants' experiences of taking part in the PHP trial highlighted the influence and power dynamics at play within South Asian family structures. Some participants noted about the resistance from their husbands or other family members resisting their participation in the program or limiting their ability to engage fully and how they engaged with the trial. These complex decision-making process that unfolds beyond the individual based on the power dynamics illustrate broader sociocultural factors, including cultural norms, gender roles, and familial authority are structured within families, often, men or elders may hold more power than women or younger members (18, 19)."
10. The limitations section could be expanded to include:<br>• Lack of representation from those who disengaged.<br>• Prior relationships between facilitators and researchers.<br>• Absence of long-term outcome data (beyond self-reported experiences).	Thank you for this suggestion. We have now expanded on the limitations section to highlighted that:<br>The lack of representation from those who disengaged are not reflected in the findings and this could bias results toward those who had positive experience on (P.23), this now reads as:<br><br>"An important limitation to note is that of the 13 PHP trial participants who withdrew from the trial were invited to take part in the interviews, only two agreed, and 11 declined or were unavailable to take part in the interview; therefore, their experiences are not reflected in the findings. This has implications for potentially biased results that favour those who have had positive experiences."

	As highlighted in your comment on point 2, we have addressed this in the Reflexivity and Researcher Positionality section om (P. 24), his now reads as:<br><br>"The research team for this study involved mainly female researchers of South Asian backgrounds and qualitative research, some of whom shared cultural and linguistic backgrounds with participants they interviewed. It is possible that this shared identity supported rapport-building and facilitated open discussion, particularly on sensitive topics such as perinatal mental health. Counteracting this, it may also have influenced how participants chose to present their experiences, and presented an element of bias by providing culturally acceptable narratives of PHP.<br>As mentioned in the earlier text, a small number of PHP facilitators were known to members of the research team prior to the study. To manage this, we explicitly acknowledged professional boundaries at the outset of the interviews and maintained a neutral, non-judgmental stance during data collection. The team acknowledged that these prior relationships might have influenced responses from PHP facilitator participants, which could have encouraged openness due to trust; however, this could have also introduced the possibility of social desirability bias. These influences were discussed during the analysis to ensure transparency and reflexivity in interpretation."<br><br><br>Thank you for highlighting the important point regarding the long-term outcome. We have now included more details on the limitations of this on p. 23, which now reads as:<br><br>"This qualitative study captures the short-term, self-reported experiences of the participants, which provide valuable insights into their immediate perceptions of PHP intervention. Despite these insights, we cannot be sure that the reported benefits were sustained over time

	or translated into meaningful long-term changes in behaviour, wellbeing, or family dynamics, without long term follow-up. To assess the durability and broader impact of the PHP, future research incorporating longitudinal designs and objective outcome measures would address this understanding."
11. The manuscript would benefit from more clearly stating its original contribution. How does PHP differ from other culturally adapted CBT interventions (e.g., MOMCare)? What does this study add to the knowledge base on group CBT among ethnic minority perinatal populations?	Thank you for this suggestions. We have highlighted the PHP unique contributions and significance of our work and how it builds on existing evidence on (P.22):<br><br>"This study makes an important contribution to the literature on culturally adapted CBT interventions for perinatal populations. While previous programs such as MOMCare have demonstrated the value of tailoring CBT to address cultural and contextual needs, PHP differs in several key respects. The PHP intervention was adapted in collaboration with South Asian women and facilitators to take into account the cultural and linguistic resonance that extends beyond surface-level adaptations. Second, PHP was delivered in a group format, which not only fostered peer support but also reflected collectivist values common in South Asian family systems.<br><br>This qualitative study provides insights into how cultural norms, family dynamics, and social expectations shape engagement with perinatal mental health care. By capturing the perspectives of both PHP trial participants and PHP facilitators, we are able to understand the knowledge base on group CBT among ethnic minority perinatal populations and highlight the importance of community-driven adaptation."
12. Consider briefly discussing the scalability of PHP, especially since remote adaptation is mentioned (p. 22, lines 568–575), but not developed.	Thank you for this suggestions. These are important points raised, and they are covered in more detail in the Lancet paper (Husain N, Lunat F, Lovell K, Miah J, Chew-Graham CA, Bee P, et al. Efficacy of a culturally adapted, cognitive behavioural therapy-based intervention for

	postnatal depression in British south Asian women (ROSHNI-2): a multicentre, randomised controlled trial. The Lancet. 2024;404(10461):1430-43) about the main trial. Due to the limited words counts for the manuscript, we have included a reference to this for readers to explore further, and we are in the process of submitting a paper that discusses the remote delivery of the PHP intervention in more detail and on the scalability and we have submitted a grant application to explore the scalability of PHP via remote delivery.
13. Minor or Technical Issues	
• Article title: The current title („Positive Health Programme for postnatal depression women...") contains a grammatical error – I suggest changing it to: „Positive Health Programme for women with postnatal depression..." or „...for South Asian women with postnatal depression".	Thank you for highlighting this, we have corrected this as follows:<br><br>"Positive Health Programme for British South Asian Women with postnatal depression: a multi-perspective qualitative study" (P.1)
• Page 7, line 202: the authors write „...they were first translated into English and then transcribed verbatim" – it's unclear who did the translations and how their quality was ensured.	In response to your comment 3, we have now provided more clarity and detail on the translation process, including the measures taken to maintain quality and the individuals involved on (P.7)
• Page 10, lines 239–240: In the sentence „Participant experiences were systematically charted to align...", it would be helpful to clarify what „systematically charted" specifically refers to (e.g., NVivo matrix, manual charting, etc.)	Thank you for this question:<br>The approach used for deductive coding was guided by the Theoretical Framework of Acceptability (TFA), for a transparent and theoretical structure for mapping themes. As we used thematic analysis to inductively capture participants' voices, while deductive coding ensured alignment with established domains of acceptability. NVivo software was used to manage transcripts, codes, and memos, enabling systematic organisation and traceability of analytic decisions. For non-English interviews, translation into English was followed by transcription and then cross-checking within the team to ensure accuracy and preserve meaning. We have included more information on this.

	The process followed for thematic analysis and coding process was as below:<br>Initial coding: Two researchers (RM, ZA) independently read transcripts line by line and applied inductive codes to capture salient concepts.<br>Code refinement: New codes were developed iteratively; overlapping codes were merged, and distinctions were clarified.<br>Collaborative discussion: The coding framework was discussed in regular meetings with the wider analysis team (SA, JB, ZA, RM, DS), allowing comparison of interpretations and resolution of discrepancies.<br>Theme development: Codes were grouped into categories and then developed into themes. Themes were subsequently mapped onto the domains of the Theoretical Framework of Acceptability (TFA).<br>Saturation: Recruitment and analysis continued until no substantially new codes or insights were emerging across interviews, which we interpreted as thematic saturation.<br><br>We have added these details on (P.8) and read as:<br><br>“An initial thematic analysis was conducted by SA, JB, ZA, RM, followed by the use of TFA (12). Thematic analysis (14) was carried out to explore and familiarise with the data by two qualitative researchers (RM, DS) who read the transcripts to identify key themes relevant to the study's aims. Two researchers (RM, ZA) independently read transcripts line by line and applied inductive codes to capture key concepts. New codes were developed iteratively; overlapping codes were merged, and distinctions were clarified. The coding framework was discussed in regular meetings with the wider analysis team (SA, JB, ZA, RM, DS), allowing comparison of interpretations and resolution of discrepancies. Codes were grouped into categories, developed into themes and then these themes were subsequently

	mapped onto the domains of the Theoretical Framework of Acceptability (TFA). Recruitment and analysis continued until no substantially new codes or insights were emerging across interviews, which we considered as thematic saturation."
• Page 22, lines 607–610: Already noted earlier – the familiarity between researchers and facilitators should be more fully addressed, as it may have influenced data collection (e.g., expectancy effect, limitations in openness, etc.).	In response to your comments 8, 9, and 10, we have addressed the points raised with the manuscript and responded to the comments above.

Reviewer: 1

If you have selected 'Yes' above, please provide details of any competing interests.: Not applicable

Reviewer: 2

If you have selected 'Yes' above, please provide details of any competing interests.: not applicable
